# Determining Antioxidant Activities of Lactobacilli Cell-Free Supernatants by Cellular Antioxidant Assay: A Comparison with Traditional Methods

**DOI:** 10.1371/journal.pone.0119058

**Published:** 2015-03-19

**Authors:** Jiali Xing, Gang Wang, Qiuxiang Zhang, Xiaoming Liu, Zhennan Gu, Hao Zhang, Yong Q. Chen, Wei Chen

**Affiliations:** 1 State Key Laboratory of Food Science and Technology, School of Food Science and Technology, Jiangnan University, Wuxi, China; 2 Synergistic Innovation Center for Food Safety and Nutrition, Wuxi, China; University of Central Florida, UNITED STATES

## Abstract

Antioxidant activity of lactic acid bacteria is associated with multiple health-protective effects. Traditional indexes of chemical antioxidant activities poorly reflect the antioxidant effects of these bacteria in vivo. Cellular antioxidant activity (CAA) assay was used in this study to determine the antioxidant activity of cell-free supernatants (CFSs) of 10 *Lactobacillus* strains. The performance of the CAA assay was compared with that of four chemical antioxidant activity assays, namely, DPPH radical scavenging, hydroxyl radical scavenging (HRS), reducing power (RP), and inhibition of linoleic acid peroxidation (ILAP). Results of the CAA assay were associated with those of DPPH and ILAP assays, but not with those of RP and HRS assays. The inter- and intra-specific antioxidant activities of CFS were characterized by chemical and CAA assays. *L*. *rhamnosus* CCFM 1107 displayed a high antioxidative effect similar to positive control *L*. *rhamnosus GG* ATCC 53103 in all of the assays. The CAA assay is a potential method for the detection of antioxidant activities of lactobacilli CFSs.

## Introduction

Increasing scientific evidence suggests that oxidative stress is involved in the pathogenesis of various disorders and diseases, such as alcohol-induced liver injury, non-alcoholic fatty liver disease, ageing, and cancer [1–4]. Oxidative stress is a result of an imbalance between production and elimination of reactive oxygen species (ROS) and free radicals, which are primarily removed by the endogenous antioxidant defense system [5]. Consumption of antioxidants, which can quench free radicals and ROS, may be beneficial to human health. Synthetic antioxidants are effective in slowing oxidation, but pose concerns in regard to the safety and toxicity of the antioxidants [6,7].

Lactic acid bacteria (LAB), which are widely used in the food industry, are generally recognized as safe [8]. LAB, particularly lactobacilli, have recently received increasing attention because of their specific role in maintaining human health and in decreasing the risk of ROS accumulation [9,10]. The benefits of lactobacilli have been consistently supported by epidemiological reports which indicate that lactobacilli are associated with a reduced risk of developing chronic diseases [11,12]. These abilities are mainly focused on viable LAB and cell-free extracts [13,14]. Furthermore, cell-free supernatant (CFS) is a well-known good source of antioxidants [15,16]. Bing et al. suggested that culture supernatant of *Lactobacillus acidophilus* contains anti-ulcer and anti-oxidative metabolites [17]. Wang et al. reported that *Lactobacillus rhamnosus* GG culture supernatant ameliorated acute alcohol-induced intestinal permeability and liver injury [18]. Shen et al. found that CFS of *Bifidobacterium animalis* 01 exhibited strong antioxidant activities (AAs) [19]. Wang et al. discovered that CFSs from 35 LAB exhibit stronger AAs than bacterial suspensions and cell-free extracts [20].

Chemical AA assays are currently available to study AAs of CFS. Commonly used chemical assays include DPPH radical scavenging, hydroxyl radical scavenging (HRS) method, reducing power (RP) and inhibition of linoleic acid peroxidation (ILAP) [19,21,22]. These assays depend on the capacity of antioxidants to quench and/or reduce free radicals. However, these chemical assays present several limitations. For instance, the DPPH assay is a fast and simple way to determine AA, however, this method does not consider certain parameters in complex cell environments, such as bioavailability and membrane permeability. Moreover, the DPPH assay is affected by light intensity, oxygen concentration, and solvent type [23]. In addition, the mechanisms of antioxidants are not only by scavenging free radicals, but also by inhibiting production of free radicals and improving levels of endogenous antioxidants [24]. Therefore, the traditional indexes of chemical antioxidant capacity poorly reflect the antioxidant effects associated with a particular sample in vivo [25].

Although studies involving animal models and humans are more appropriate than in vitro studies, they are expensive and time consuming [25]. Meanwhile, cellular AA (CAA) assay is an extremely attractive intermediate testing method to support antioxidant research prior to animal studies and human clinical trials [26]. This method shows high physiological quality in AA measurement, thus, it has been applied to study several natural product extracts, foods, and dietary supplements [25]. Data obtained from a cell-based assay may be comprehensively interpreted given the multiple health-protective effects of lactobacilli CFS.

The present study aims (i) to evaluate the toxicity of CFS using HepG2 cell line, (ii) to apply CAA assay to detect AAs of CFS, (iii) to analyze the difference of inter and intra-specific of CFS by comparing the antioxidant activities of 10 *Lactobacillus* strains from seven species which are used as probiotic bacteria, and (iv) to study the correlations between chemical and CAA assays to provide theoretical guidance in rationally screening LAB.

## Materials and Methods

### Bacterial Strains and Culture Conditions

All *Lactobacillus* strains used in this study are listed in Table 1, including *L*. *rhamnosus GG* ATCC 53103 (LGG), which was used in all of experiments as a reference strain [12,27]. *L*. *acidophilus*, *L*. *fermentum*, *L*. *rhamnosus*, *L*. *casei*, *L*. *plantarum* and *L*. *reuteri* are known species that display AAs [12,28–32]. *L*. *farciminis* which is rarely reported to exhibit AA was also adopted as a reference strain. These strains were maintained as frozen stocks (−80°C) in deMan, Rogosa, and Sharpe (MRS) broth (Hopebio Company, Qingdao, China) supplemented with 30% (v/v) glycerol. All strains were consecutively transferred at least three times using 1% (v/v) inoculum in MRS broth at 37°C for 20 h prior to use.

**Table 1:** Lactic acid bacteria used in this study.

*Lactobacillus* Strain	Characteristics	Source or Reference
*L*. *rhamnosus GG* ATCC 53103	Healthy human intestinal flora	Valio Ltd., Helsinki, Finland
*L*. *rhamnosus* CCFM-JU 1107	Pickles	CCFM-JU*
*L*. *rhamnosus* CCFM-JU 7469	Pickles	CCFM-JU
*L*. *casei* 2W	Commercial yoghurt	TCBD*
*L*. *plantarum* CCFM-JU 8661	Pickles	CCFM-JU
*L*. *reuteri* CCFM-JU 14	Pickles	CCFM-JU
*L*. *acidophilus* CCFM-JU 137	Human feces	CCFM-JU
*L*. *farciminis* CCFM—JU 419	Fish tea	CCFM-JU
*L*. *fermenti* CCFM-JU 381	Old leavened dough	CCFM-JU
*L*. *fermenti* CCFM-JU 424	Acid kidney bean	CCFM-JU

CCFM-JU*, Culture Collection of Food Microorganisms of Jiangnan University (Wuxi, China);

TCBD*, Technology Center of Bright Dairy and Food Co., Ltd. (Shanghai, China).

### Solution Preparation

A: Linoleic acid emulsion (20 mL) was prepared by mixing 0.1 mL of linoleic acid (Sigma—Aldrich, St. Louis, MO), 0.2 mL of Tween 20, and 19.7 mL of deionized water, and the emulsion was stored in the dark.

B: A 200 mM stock solution of 2’, 7’-dichlorofluorescin diacetate (DCFH-DA) (Sigma—Aldrich, St. Louis, MO) in dimethyl sulfoxide (Sigma—Aldrich, St. Louis, MO) was prepared, aliquoted, and stored at -20°C.

C: A 200 mM stock solution of 2, 2-azobis (2-amidinopropane) dihydrochloride (ABAP) (Sigma—Aldrich, St. Louis, MO) in Hanks’ Balanced Salt Solution (HBSS) (Sigma—Aldrich, St. Louis, MO) was prepared and stored at -40°C.

### Preparation of lactobacilli CFS

Lactobacilli CFSs were prepared as described by Chen and Wang [12,18] with slight modification. Cultures of the 10 *Lactobacillus* strains were adjusted to approximately 10^9^ CFU/mL. Subsequently, the aliquots of the culture were transferred to 5 mL polypropylene tubes and centrifuged (10,000*g*, 10 min, 4°C). The pH value of the supernatant was continuously neutralized with 1 M NaOH [33]. The resulting supernatant was filtered (0.22 μm pore size). LGG CFS was used as a positive control. MRS broth medium (pH 7.0, filtered with a 0.22 μm pore size filter) served as a negative control.

### Chemical Assays to Determine AAs of Lactobacilli CFSs

#### DPPH Radical Scavenging Activity Assay

The scavenging effect of the CFSs of 10 *Lactobacillus* strains on the free radical DPPH was measured in accordance with the slightly modified method of Lin and Chang [28]. A Sample (CFS, MRS broth, 1 mL) and a freshly prepared DPPH solution (0.2 mM, 1 mL, Sigma—Aldrich, St. Louis, MO) were mixed. The mixture was vigorously shaken and left to react for 30 min in the dark at room temperature. The control sample contained deionized water instead of the sample solution. The scavenged DPPH was then monitored by determining the absorbance at 517 nm using SpectraMax M5 microplate reader (Molecular Devices, Sunnyvale CA). The radical scavenging activity was quantified as units/mL (U/mL) by using following formula [34]:

$$DPPH\;activity\;(U/mL)=\left(ABS_{C}-ABS_{S}\right)/S\times 100$$

where *ABS*_*C*_ and *ABS*_*S*_ are the absorbance of the control and test samples at 517 nm, respectively, and *S* is the volume (mL) of the sample.

#### HRS Activity of Lactobacilli CFSs

The HRS activity of the CFSs was analyzed as previously described modified method [13,19]. A sample (CFS, MRS broth, 1 mL), 1, 10-phenanthroline (2.5 mM, 1 mL; Sigma—Aldrich, St. Louis, MO), PBS (pH 7.4, 1 mL), and FeSO_4_ (2.5 mM, 1 mL) were mixed. The reaction was initiated by adding H_2_O_2_ (20 mM, 1 mL) and incubating at 37°C for 90 min. HRS activity was monitored by identifying the increase in absorbance at 536 nm by using an SpectraMax M5 microplate reader (Molecular Devices, Sunnyvale CA). HRS activity was calculated using the following equation:

$$HRS\;activity\;(\%)=(A_{S}-A_{C})\;/(A_{b}-A_{C})\times 100\%$$

where *A*_*S*_ is the absorbance of the sample, *A*_C_ is the absorbance of the control solution (deionized water was used instead of the sample at the same amount), and *A*_*b*_ is the absorbance of the solution without samples and H_2_O_2_.

#### RP of Lactobacilli CFSs

The CFS reducing activity of the CFSs was determined as described by Lin and Yen [35] with slight modification. A sample (CFS, MRS broth, 0.5 mL) was briefly mixed with potassium ferricyanide (1%, 0.5 mL) and PBS (pH 6.6, 0.5 mL). Subsequently, the mixture was heated at 50°C for 20 min and allowed to cool. Upon cooling, 0.5 mL of 10% trichloroacetic acid (TCA) was added to the mixture and then centrifuged at 3000g for 5 min. The upper layer (1 mL) was mixed with ferric chloride (0.1%, 1 mL) and allowed to react for 10 min. The absorbance of the mixture was obtained at 700 nm by using an SpectraMax M5 microplate reader (Molecular Devices, Sunnyvale CA). Higher absorbance of the mixture indicated higher reducing activity. The reducing activity of cysteine served as the standard.

#### ILAP of Lactobacilli CFSs

The anti-lipid peroxidation activity of the CFSs was assessed by using the thiobarbituric acid (TBA) method [22] with slight modifications. PBS (pH 7.4, 0.5 mL), linoleic acid emulsion (1 mL), FeSO_4_ (0.01% w/v, 0.2 mL), ascorbic acid (0.02%, w/v, 0.2 mL), and sample (CFS, MRS broth, 0.5 mL) were mixed and then incubated at 37°C for 12 h. Subsequently, 2.0 mL of the reaction mixture was mixed with butylated hydroxytoluene (0.4% w/v, 0.2 mL), TCA (4% w/v, 0.2 mL) and TBA (0.8% w/v, 2 mL). The mixture was incubated at 100°C for 30 min and then allowed to cool, 2 mL of chloroform was then added for extraction. The upper extract was obtained and absorbance was determined at 532 nm by using an SpectraMax M5 microplate reader (Molecular Devices, Sunnyvale CA). The samples were substituted with deionized water in the control group. The inhibition rate was calculated by using the following equation:

$$Inhibition\;effect\;(\%)=(1-A_{S}/A_{C})\times 100\%$$

where *A*_S_ is the absorbance of the sample, and *A*_C_ is the absorbance of control solution that sample solution, in which the sample solution was replaced with same amount of deionized water.

### Assessment of Antioxidant Effects of Lactobacilli CFSs using CAA Assay

#### Cell Culture

Human hepatocellular carcinoma HepG2 cells (Cell Bank of the Type Culture Collection of the Chinese Academy of Sciences, Shanghai, China) were grown in HyClone Dulbecco’s modified Eagle’s medium (DMEM) with high glucose (GE Healthcare, USA) supplemented with 10% fetal bovine serum (Gibco, Grand Island, NY, USA), penicillin (100 U/mL), streptomycin (100 μg/mL) (Sigma—Aldrich, St. Louis, MO), and 10 mM 4(2-hydroxyethyl)-1-piperazineethanesulfonic acid (HEPES) (Sigma—Aldrich, St. Louis, MO). The cells were maintained at 37°C in an incubator with 5% CO_2_. The cells used in this study were at passage 10 to 20.

#### Cell Cytotoxicity Assay

Cytotoxicity of the lactobacilli CFSs was measured according to the modified methylene blue assay [36]. Briefly, HepG2 cells were seeded at a density of 4×10^4^ cells/well on a 96-well microplate in 100 μL of DMEM for 24 h at 37°C. After washing with PBS, HepG2 cells were treated with 100 μL of the sample (CFS, MRS) or deionized water (control) for up to 24 h at 37°C. To assess cell viability, the cells were washed with PBS and then incubated with 50 μL/well methylene blue (98% HBSS, 0.67% glutaraldehyde, and 0.6% methylene blue) for 1 h at 37°C. After the incubation, the cells were washed with PBS until the PBS was clear, and 100 μL/well elution (49% PBS, 50% ethanol, and 1% acetic acid) was then added, the microplate was then placed on a table oscillator (Thermomixer Comfort, Eppendorf AG, Hamburg, Germany) for 20 min. The absorbance was obtained at 570 nm by using an SpectraMax M5 microplate reader (Molecular Devices, Sunnyvale CA). Different samples were compared with the control. A CFS with more than 10% less absorbance than the control, was considered cytotoxic [37].

#### CAA Assay

CAA assay was used to evaluate the lactobacilli CFSs as previously described [26,38,39]. Briefly, HepG2 cells, the most commonly used in CAA assay [26], were seeded at a density of 6×10^4^ cells/well on a black 96-well microplate (with transparent bottoms) in 100 μL of DMEM for 24 h at 37°C. After rinsing with PBS, HepG2 cells were treated with 100 μL of the sample (CFS, MRS broth), which includes 25 μM of DCFH-DA for up to 1 h at 37°C. The cells were washed with PBS and then treated with 100 μL of 600 mM ABAP solution. Fluorescence was obtained using an SpectraMax M5 microplate reader (Molecular Devices, Sunnyvale CA) for 13 cycles at 5 min intervals (λex = 485 and λem = 538). After blank subtraction from the fluorescent readings, the area under the curve of fluorescence versus time was integrated to calculate the CAA value of each sample as follows:

CAA(unit)={1-(∫SA/∫CA)}×100

Where SA is the area of the sample, and CA is the integrated area in the control curve.

### Statistical Analysis

All of the tests were performed in triplicate. Data were presented as mean ± standard deviation (SD). One-way ANOVA was performed with SPSS (Version 13.0, SPSS Inc., Chicago, IL), followed by Fisher’s least significant difference, to verify significant differences between samples. The results were considered significant when *p* < 0.05. The Pearson correlation test was conducted to determine correlation between variables.

## Results and Discussion

### Chemical AA Assay of Lactobacilli CFSs

#### Radical Scavenging Activities

**Fig 1:**
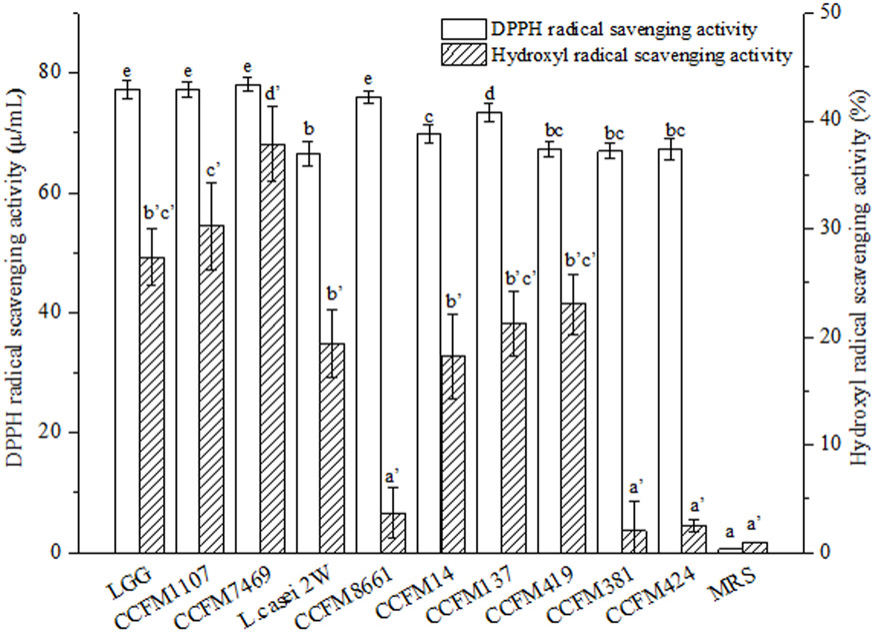
DPPH and HRS activities of the CFSs (mean ± SD, n = 3). MRS broth control sample without *Lactobacillus*. The letters a, b, c, d, and e indicate statistically significant difference at *p* < 0.05 within each row comparison between the DPPH radical scavenging groups. The letters a’, b’, c’, d’, and e’ indicate statistically significant difference at *p* < 0.05 within each row comparison between the HRS groups. Bars with no common letters are significantly different (*p* < 0.05).

Results of two radical scavenging methods (i.e., HRS and DPPH) showed that the 10 lactobacilli CFSs can inhibit the formation of the two radicals (Fig. 1). The CFSs of the 10 lactobacilli CFSs showed remarkably weaker HRS activity than DPPH radical scavenging activity. Furthermore, the AAs of the 10 lactobacilli CFSs were higher than that of MRS broth in scavenging the DPPH radical. This finding is consistent with the report of Shen et al. [19]. In addition, the HRS method provided similar results on the AAs of the CFSs (Fig. 1). Significant differences in AA values were observed between MRS broth and CFSs, except for CCFM 8661, CCFM381, and CCFM 424 in scavenging hydroxyl radicals (Fig. 1, *p* < 0.05). Both the HRS and DPPH methods indicated that the CFSs of CCFM 1107 and CCFM 7469 exhibited strong AAs.

The CFSs of CCFM8661 exhibited strong DPPH radical scavenging activities (75.94 ± 1.05 U/mL) that were not significantly different from that of the positive control LGG (77.29 ± 1.51 U/mL) (*p* > 0.05). However, CCFM8661 demonstrated significantly weaker HRS activity than LGG in scavenging hydroxyl radical (*p* < 0.05). Furthermore, the effect of *L*. *casei* 2W on DPPH and hydroxyl radical contradicted that of CCFM8661. Intact cells and intracellular cell-free extracts from *L*. *casei* subsp. *casei* SY13 and *L*. *delbrueckii* subsp.*bulgaricus* LJJ also differ in DPPH and HRS activities [13]. The different mechanisms involved in the radical-antioxidant reactions may explain the different in scavenging potentials of the compounds [40]. Moreover, the DPPH and hydroxyl radical activities of both *L*. *rhamnosus* CCFM 1107 and CCFM 7469 were significantly stronger than those of *L*. *fermenti* CCFM381 and CCFM 424 (*p* < 0.05). Liu et al. [8] reported that 12 *Lactobacillus* strains exhibit varying capabilities in DPPH radical scavenging. Therefore, a certain degree of inter-specific difference in radical scavenging activities could exist among the 10 tested *Lactobacillus* strains.

#### RP of Lactobacilli CFSs

**Fig 2:**
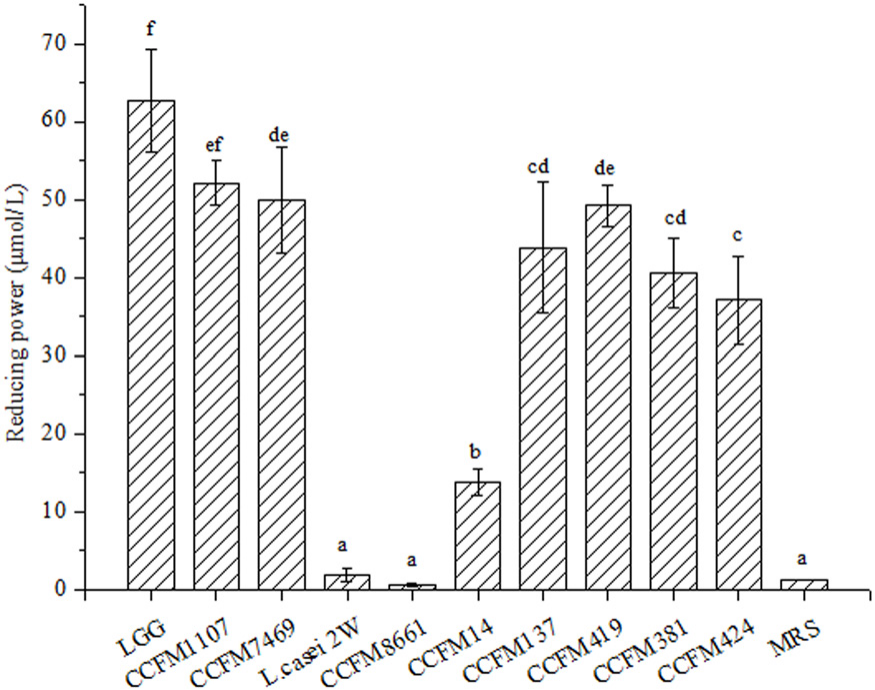
RP values of the CFSs (mean ± SD, n = 3). MRS control sample without lactobacilli. RP values are expressed as μM of cysteine. Different letters indicate statistically significant differences at *p* < 0.05. Bars with no common letters are significantly different (*p* < 0.05).

An earlier report showed that AAs and RP are directly related [41]. The RP of lactobacilli CFS is based on kinetics of the reduction of Fe^3+^ to Fe^2+^ to prevent the oxidation reaction and control transition metal ions [41]. The RP activities of lactobacilli CFS are shown in Fig. 2. The CFSs of LGG, CCFM1107, CCFM7469, CCFM14, CCFM137, CCFM419, CCFM381, and CCFM424 showed significantly higher RP values than that of MRS broth (*p* < 0.05). Meanwhile, the CFSs of *L*. *casei 2W* and CCFM 8661 increased, but not significantly, compared with that of MRS broth (*p* > 0.05). In all of the tests, CCFM1107 showed a high reducing activity that was close to that of the positive control LGG and equivalent to that of 62.68 μM *L*-cysteine. The results are consistent with the findings of a previous report [8], which revealed that 12 strains show varied abilities in reducing RP activity and that *L*. *acidophilus* BCRC 14079 displays six-fold stronger activity than *B*. *infantis* BCRC 14602. Therefore, an inter-specific difference exists in the RP of the test CFSs.

#### ILAP of Lactobacilli CFSs

**Fig 3:**
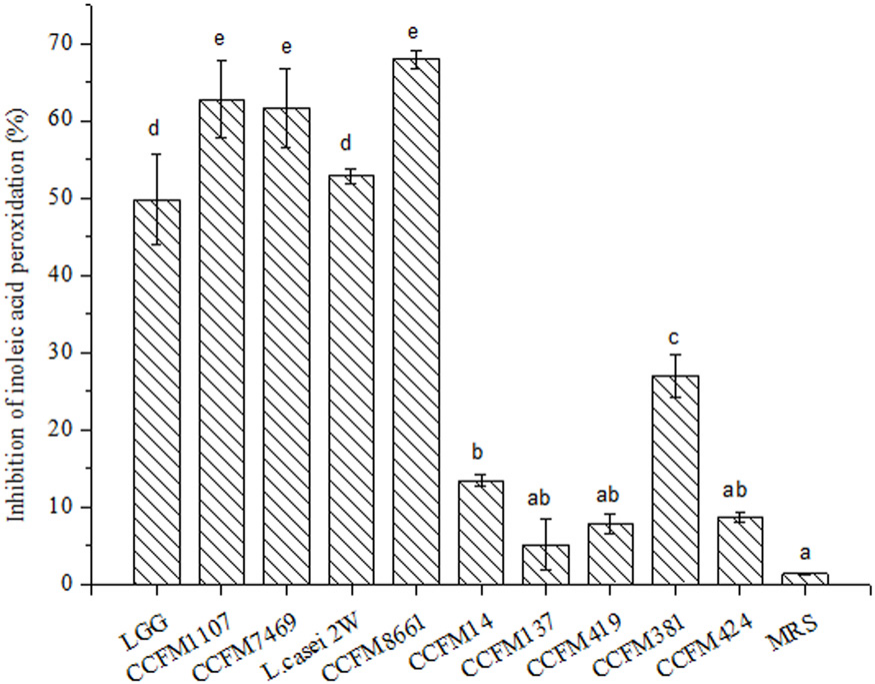
ILAP of the CFSs (mean ± SD, n = 3). MRS control sample without lactobacilli. Different letters mean statistically significant differences at *p* < 0.05. Bars with no common letters are significantly different (*p* < 0.05).

ILAP is commonly used to analyze AAs. Linoleic acid has been used as a source of unsaturated fatty acids [42]. In the present study, the AAs of lactobacilli CFSs were measured by ILAP assay. As shown in Fig. 3, the inhibitory rates of test lactobacilli CFSs on linoleic acid peroxidation ranged from 5.1 ± 0.22% to 67.93 ± 1.22% (Fig. 3). The inhibitory rates of the lactobacilli CFSs were significantly higher than those of MRS broth (*p* < 0.05), except for CCFM137, CCFM419, and CCFM424 (*p* > 0.05). The CFSs of CCFM1107, CCFM7469, and CCFM 8661 showed high inhibitory effects on ILAP compared with the positive control LGG (*p* > 0.05). Meanwhile, *L*. *rhamnosus* CCFM1107 and CCFM7469 demonstrated significantly stronger activities on ILAP than did *L*. *fermenti* CCFM381 and CCFM424. These observations indicated an inter-specific difference in ILAP among the lactobacilli CFSs. Moreover, the higher inhibitory effect of CCFM381 CFS than of CCFM424 CFS indicated an intra-specific difference in ILAP between these two CFSs. These results agree with the findings of an earlier report [42], which revealed that the inhibitory rates of six strains of *L*. *acido*p*hilus* on ILAP range from 34.9% to 46.3%.

#### Correlations among the Four Chemical AA Assays

Results indicated that the lactobacilli CFSs, which were measured by using chemical methods, exhibited certain antioxidant properties. Among the tested CFSs, LGG and CCFM1107 had the highest AA in all of the assays. In addition, lactobacilli CFSs exerted different AAs in various chemical antioxidant models. For example, the CFS of CCFM8661, CCFM7469, and *L*.*casei* 2W displayed different AA values in radical scavenging, RP, and ILAP. In a previous study, Zhu et al. [43] found that the water extracts of okara koji and the water extract of soybean koji show different AAs according to different in vitro antioxidant models. In this case, four chemical assays were used and compared to determine the antioxidant capacities of the CFSs, and their correlations are shown in Table 2. A significant association was found between scavenging of DPPH and hydroxyl radicals (*r* = 0.511, *p* < 0.01, n = 11). Meanwhile, the DPPH radical scavenging of the CFSs correlated significantly with ILAP (*r* = 0.604, *p* < 0.01, n = 11). HRS also had a significant association with RP (*r* = 0.494, *p* < 0.01, n = 11). However, ILAP showed no significant correlation with HRS and RP because of the diverse chemical aspects of potential antioxidant compound(s) explored [25]. The lack of correlation between results obtained through these different assays can be attributed to reasons related to instrument limitations, mechanisms, endpoint, quantification method, and biological relevance [44]. Therefore, the use of a single chemical method to screen strains with high AAs is difficult due to the different mechanisms of CFSs in vivo.

**Table 2:** Correlations among the four chemical antioxidant activity assays.

Correlation coefficient	DPPH radical scavenging	HRS	RP	ILAP
DPPH radical scavenging	1			
HRS	0.511**	1		
RP	0.377	0.494**	1	
ILAP	0.604**	0.273	−0.012	1

* *p* < 0.05

** *p* < 0.01

### CAA Assessment of the AAs of Lactobacilli CFSs

#### Cell Cytotoxicity

**Fig 4:**
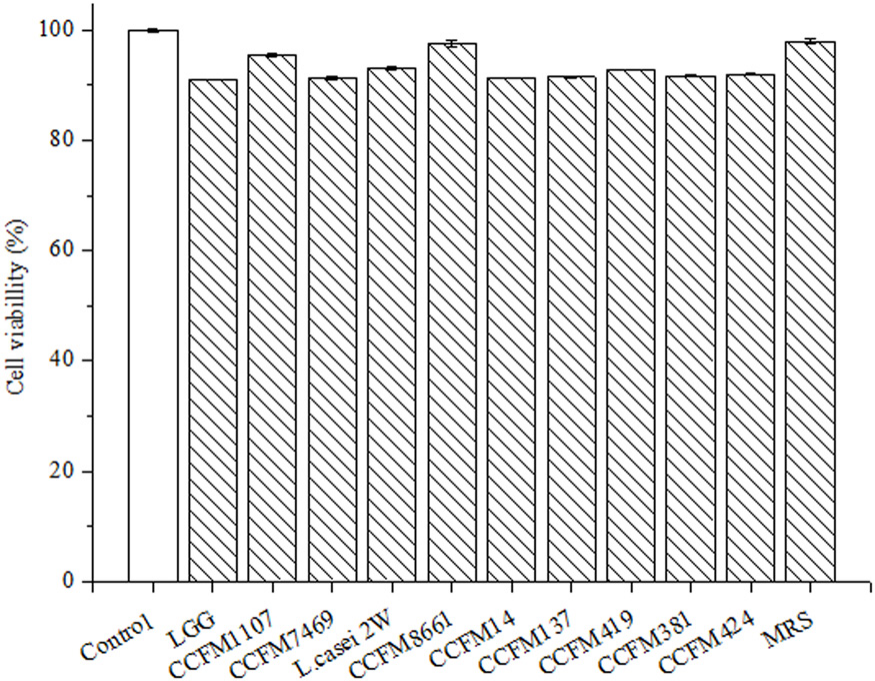
Cytotoxicity of the CFSs on human hepatocellular carcinoma HepG2 cells. Negative control was untreated cells (mean ± SD, n = 3).

Modified methylene blue assay is a typically fast and easy method of detecting cell death. Studies have shown that HepG2 cells could be used to demonstrate cell toxicity [36,45]. However, the toxicity of CFS on HepG2 cells has not been evaluated. In the present study, CFS and MRS broth were used to react with HepG2 cells and methylene blue was used to stain the HepG2 cells. Results showed that incubation with CFS and MRS broth inhibited less than 10% of HepG2 cells (Fig. 4). This result indicates that both CFS and MRS broth do not show significant cytotoxicity to HepG2 cells after 24 h incubation [37]. Previous research showed that feijoada whole meal, apple extracts, and vegetables inhibit less than 10% of HepG2 cells [39,46,47]. Therefore, we deduced that the CFSs are not cytotoxic, which is a typical observation for such functional products. Moreover, Maudsdotter et al. [48] found that the CFS of LGG can reduce cell cytotoxicity caused by *Streptococcus pyogenes* by producing lactic acid. Li et al. [49] showed that the CFSs of *L*. *acidophilus* not only display non- cytotoxicity, but also stimulate proliferation of embryonic, endothelial, and inflammatory cells in vivo. These observations suggested that the CFSs of the *Lactobacillus* strains are non-cytotoxic.

#### CAA Assay

**Fig 5:**
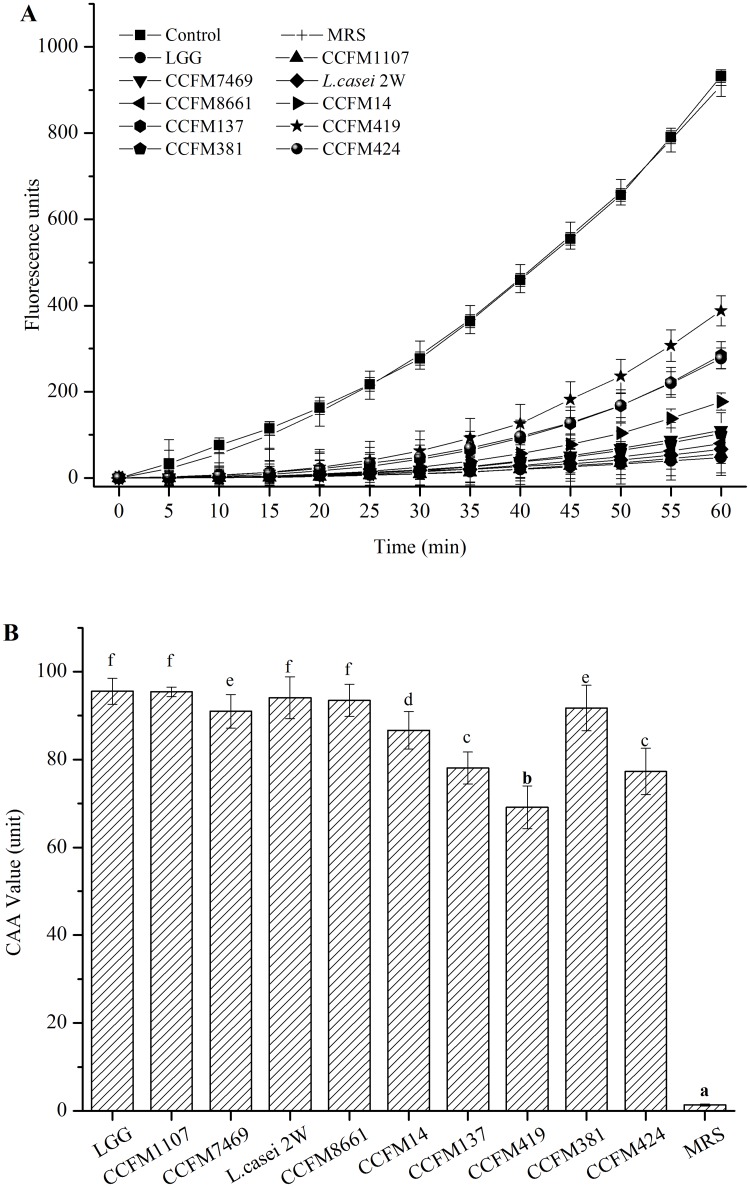
AAs of the CFSs evaluated by the CAA method. (A) Peroxyl radical-induced oxidation of DCFH to DCF in HepG2 cells, and inhibition of oxidation by CFS and MRS (mean ± SD, n = 3). (B) CAA units of CFS and MRS broth. CAA unit was calculated as the difference in the area under the curve between the tested samples and control wells. Data represent the mean ± SD values that were obtained from six wells in each group. Different letters mean statistically significant differences at *p* < 0.05.

In the CAA assay developed by Wolfe and Liu [26], the ABAP-induced oxidation of dye DCFH-DA to fluorescent DCF and the fluorescence intensity of DCF were measured to represent the oxidation rate, thereby enabling the CAA assay to measure the antioxidation capacity of the antioxidants [26,50]. In the present study, we applied the CAA assay to determine the AAs of lactobacilli CFSs. The positive control showed fluorescence intensity of ABAP oxidation over time. The negative control group contained the cells without the addition of ABAP. These cells were used to illustrate the conditions devoid of any oxidation inducer. The CFSs of all 10 *Lactobacillus* strains inhibited oxidation, with higher fluorescence intensities than the negative control (*p* < 0.05) (Fig. 5A). However, these intensities were lower than that of the positive control over time, except for the MRS broth, which showed similar intensities to the positive control. The trends of inhibiting DCFH-DA to DCFH of CFS are similar to those of pure compounds, such as theacrine, sugarcane molasses, and *Crataegus azarolus* [51–53]. Puertollano et al. previously reported that concentrated supernatants from *L*. *plantarum* reduce ROS accumulation in HL-60 cells [54]. In addition, the present results showed that the ROS in HepG2 cells could be reduced by lactobacilli CFSs. Similar to other methods used for AA assessment, the CAA method reveals the total antioxidative capacity of the analyzed sample rather than the capacity of the individual components of the system. However, the CAA also differs in other aspects. It is the only method capable of predicting antioxidant response at the cellular level. It also allows the analysis of samples activity to change the redox cellular state. Moreover, the participation of different component cells is critical to develop an antioxidant response [25].

CAA units were calculated on the basis of the area under the curve of the fluorescence intensities of the CFS and ABAP-treated cells over time. A smaller area denotes higher CAA units and higher AAs of the sample [51]. All of the lactobacilli CFSs possessed significantly higher CAA values than the MRS broth (*p* < 0.05) (Fig. 5B). The cellular AA of quercetin was also determined by CAA assay in HepG2 cells (S1 Fig.) as a standard. The calculation results of CAA values (units) of the CFSs of *Lactobacillus* strains to the equivalent amount of quercetin with the same AA (μM) present more intuitive view of the AA of the CFSs of the *Lactobacillus* strains compared with this established antioxidant with known clinical efficacy (S1 Table). The CAA values of the CFSs ranged from 69.14 ± 4.87 to 95.55 ± 2.99 CAA units. The positive control LGG also exhibited a strong antioxidant property (95.55 ± 2.99 CAA units) in the CAA assay, which was stronger than that of *Crataegus azarolus* aqueous extract (79.62 CAA units in 800 μg/mL) [53]. The difference essentially depends on the bioavailability of the specific mixture of the available compounds and their synergistic interactions to yield final antioxidant responses at the cellular level [25]. CCFM1107, *L*. *casei* 2W, and CCFM8661 showed the highest AAs (95.40, 94.06, and 93.48 CAA units, respectively). No significant difference was found with LGG (*p* > 0.05). However, CCFM419 provided the lowest CAA value, which was significantly different from that of the other strains (*p* < 0.05). Consequently, the 10 *Lactobacillus* strains showed significant interspecific differences in CAA. *L*. *rhamnosus* showed a higher CAA value than that of *L*. *fermenti*. CCFM1107 and CCFM7469 originating from the same *L*. *rhamnosus*, but the two strains had significantly different CAA values (*p* < 0.05). Similar results were observed between *L*. *fermenti* CCFM381 and *L*. *fermenti* CCFM424. These observations indicate intra-specific differences of test CFSs in the CAA assays. Various researchers have reported similar findings which were obtained using different methods [19,55] that is, intra- and inter-specific differences exist for both intact bacteria and CFS.

#### Correlations between CAA and the Four Chemical AA Assays

**Fig 6:**
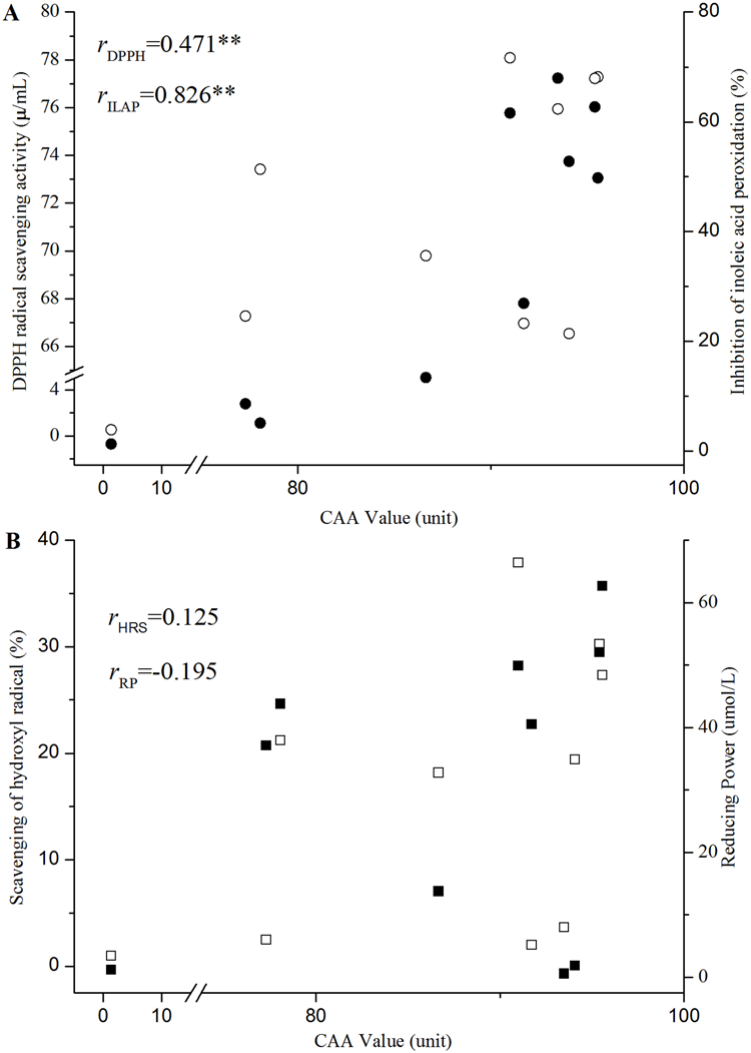
DPPH radical scavenging, HRS, RP, and ILAP values vs. CAA indexes for the different CFSs and MRS broth. (A) CAA values vs DPPH radical scavenging and ILAP indexes for CFSs and MRS broth. (B) CAA values vs HRS and RP indexes for CFSs and MRS broth. (○) represents DPPH radical scavenging values; (●) represents HRS values; (☐) represents RP values; and (■) represents ILAP values. * and ** mean statistically significant difference at *p* < 0.05 and *p* < 0.01, respectively. Abbreviations: HRS, hydroxyl radical scavenging; RP, reducing power; ILAP, inhibition of linoleic acid peroxidation; CFSs, cell-free supernatants; CAA, cellular antioxidant activity; r_DPPH_, correlation between the CAA and DPPH radical scavenging activity assay; r_ILAP_, correlation between the CAA and ILAP assay; r_HRS_, correlation between the CAA and HRS assay; and r_RP_, correlation between the CAA and RP assay.

The AA values of lactobacilli CFSs assessed with the four chemical assays did not correlate well with their abilities to inhibit the radical-mediated damage on HepG2 cells (CAA index). The CFSs of several *Lactobacillus* strains that exhibited low chemical AAs showed high responses in the CAA assay (Fig. 6). For instance, the CFSs of *L*. *casei* 2W and CCFM8661 showed weak AAs in HRS and RP, but were proven efficacious in the CAA assay. CCFM419 had low antioxidant efficacy in the CAA assay, but exhibited high HRS activity. No significant association was found among the results of the HRS, RP, and CAA assays (*r*_HRS_ = 0.125, r_RP_ = −0.195, *p* > 0.05, Fig. 6A). This observation agrees with the report of Huang et al. [56], in which the antioxidant values of Chinese bayberry obtained from ABTS, FRAP, DPPH, ORAC, and CAA assays do not correlate significantly with one another. In this case, the results are not surprising because the CAA assay monitors oxidative stress in cells that are related to cellular uptake, distribution, and metabolism of antioxidants. In chemical assays, antioxidants directly react with radicals [44]. Conversely, CCFM8661 has a high value in CAA, DPPH, and ILAP. The CAA assay revealed a significant association with DPPH and ILAP (*r*_DPPH_ = 0.471, *r*_ILAP_ = 0.826, *p* < 0.01, Fig. 6B). In addition, LGG and CCFM1107 exhibited high AAs in the four chemical assays and CAA assay, whereas MRS showed a low AA. Considering the inter- and intra-species difference among numerous bacteria strains, the AAs evaluated by CAA assay show accordance with that got by the four traditional chemical assays to some extent. For the mammalian cells engaged in CAA assay, the AAs evaluated by this assay may be more correlated with the actual situation in organisms than that got with chemical assays. Thus, the CAA assay is a potential method for the detection of AAs of lactobacilli CFSs.

## Conclusion

This study shows that lactobacilli CFSs exhibit AAs that can be assessed quantitatively in HepG2 cells. The CAA method is a relatively reliable and sensitive method compared with the four chemical assays in screening the AAs of CFS. Oxidative stress is involved in numerous chronic degenerative diseases. CFSs that show AAs can be evaluated by the CAA method as promising candidates in the prevention and control of several free radical-related disorders. Future studies will be conducted to elucidate the possible mechanisms of CFS and to verify the specific functions of CFSs screened by CAA assay in animal models or human studies.

## Supporting Information

S1 Fig Calibration curves with nonlinear fitting of AAs of quercetin evaluated by the CAA method. The CAA value was calculated based on the difference in the area under the curve between the tested samples and control wells.
(TIF)

S1 Table CAA values of the CFSs of the 10 Lactobacillus strains and MRS broth. The CAA value is expressed as an equivalent amount of quercetin (μM). Different letters indicate statistically significant differences at p < 0.05.
(DOCX)
