# Supplementary figures and images for: Determining Antioxidant Activities of Lactobacilli Cell-Free Supernatants by Cellular Antioxidant Assay: A Comparison with Traditional Methods
Source: PLoS One. 2015 Mar 19;10(3):e0119058. doi: 10.1371/journal.pone.0119058 (PMC4366247; doi:10.1371/journal.pone.0119058)

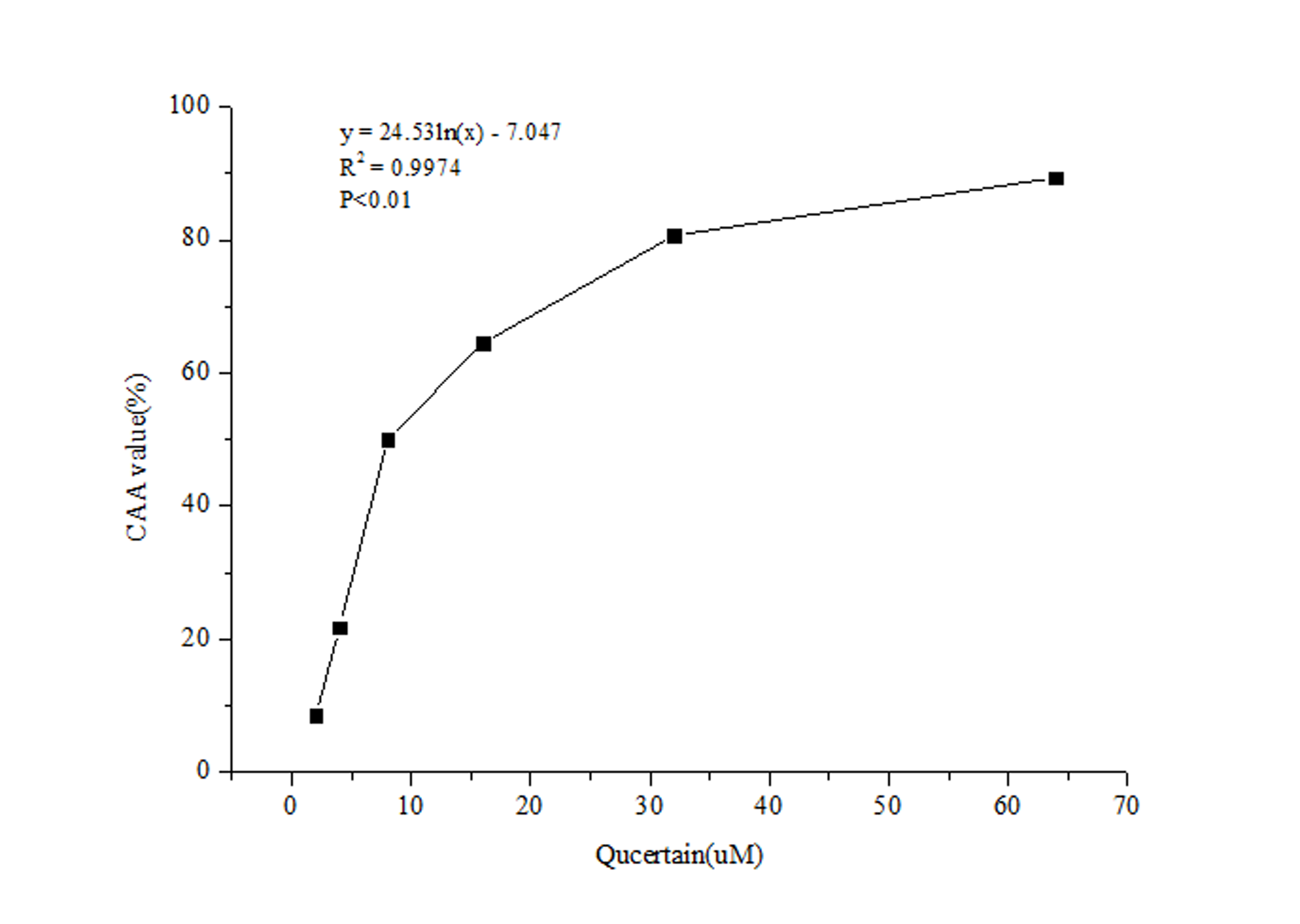

Supplement: S1 Fig — The CAA value was calculated based on the difference in the area under the curve between the tested samples and control wells. (TIF) [file pone.0119058.s001.tif]
