# Supplementary material for: Determining Antioxidant Activities of Lactobacilli Cell-Free Supernatants by Cellular Antioxidant Assay: A Comparison with Traditional Methods
Source: PLoS One. 2015 Mar 19;10(3):e0119058. doi: 10.1371/journal.pone.0119058 (PMC4366247; doi:10.1371/journal.pone.0119058)
Supplement: S1 Table — The CAA value is expressed as an equivalent amount of quercetin (μM). Different letters indicate statistically significant differences at p < 0.05. (DOCX) [file pone.0119058.s002.docx]

| *Sample* | CAA value (µM quercetin) |
| --- | --- |
| *L. rhamnosus GG* ATCC 53103 | 65.534^f^ |
| *L. rhamnosus* CCFM-JU 1107 | 65.120^f^ |
| *L. rhamnosus* CCFM-JU 7469 | 54.406^e^ |
| *L. casei* 2W | 61.669^f^ |
| *L. plantarum* CCFM-JU 8661 | 60.223^f^ |
| *L. reuteri* CCFM-JU 14 | 65.574^d^ |
| *L. acidophilus* CCFM-JU 137 | 32.115^c^ |
| *L. farciminis* CCFM –JU 419 | 22.324^b^ |
| *L. fermenti* CCFM-JU 381 | 56.036^e^ |
| *L. fermenti* CCFM-JU 424 | 31.121^c^ |
| MRS broth | 1.408^a^ |

**S1 Table.**
